# Supplementary material for: Nest Entrances, Spatial Fidelity, and Foraging Patterns in the Red Ant Myrmica rubra: A Field and Theoretical Study
Source: Insects. 2020 May 21;11(5):317. doi: 10.3390/insects11050317 (PMC7290572; doi:10.3390/insects11050317)
Supplement: Supplementary file 1 [file insects-11-00317-s001.zip › Supplementary/Table_S1.pdf]

Table S1. Summary of observation sessions performed for each colony

	Foraging experiment		Fidelity experiment	
Colony	Number of observations	Total number of observed entrances*	Number of observations	Number of active entrances
1	3	5	0**	1
2	3	8	2	4
3	1	3	0**	1
4	2	2	0**	1
5	1	6	2	5

*: Total number of active entrances recorded over the different sessions of observation.

**: Experiment of fidelity was not carried out because the nest showed only one entrance when the observation was made.
