# Supplementary material for: Nest Entrances, Spatial Fidelity, and Foraging Patterns in the Red Ant Myrmica rubra: A Field and Theoretical Study
Source: Insects. 2020 May 21;11(5):317. doi: 10.3390/insects11050317 (PMC7290572; doi:10.3390/insects11050317)
Supplement: Supplementary file 1 [file insects-11-00317-s001.zip › Supplementary/Table_S2.pdf]

Table S2. Summary of paint-marked ants per colony in the fidelity experiment

Colony	Observation session	Number of source/entrance couple observed	Total number of marked ants	Total number of marked ants observed entering an entrance	Total number of marked ants observed at a food source
2	1	4	34	42	34
2	2	3	37	44	21
5	1	4	31	72	31
5	2	3	30	97	30
