# Supplementary material for: Nest Entrances, Spatial Fidelity, and Foraging Patterns in the Red Ant Myrmica rubra: A Field and Theoretical Study
Source: Insects. 2020 May 21;11(5):317. doi: 10.3390/insects11050317 (PMC7290572; doi:10.3390/insects11050317)
Supplement: Supplementary file 1 [file insects-11-00317-s001.zip › Supplementary/Model.pdf]

Model

In this model, the ants exited the nest to navigate and look for food items on a hexagonal grid, each side of the hexagonal grid was 50-cell long, for a long diagonal of 101 cells (for an example of the results obtained on a smaller grid, see Fig. SM2 at the end of this supplement). Once an item was discovered, the successful ants came back to the nest and recruited additional nestmates towards the food source. The model was elaborated considering our experimental observations in the field (see Results section 3.1) and existing literature on the red ant *M. rubra*.

More specifically, we included the following characteristics of ants' behaviour in the agent-based simulations:

- A group of ants was available for recruitment in each nest entrance.
- Ants showed a high fidelity to their nest entrance and recruited nestmates only at this location.
- Ants showed a strong fidelity to the first food source that they exploited and favoured the shortest path leading to this food source.
- When homing back to their nest, ants were likely to move forward and to favour the shortest path leading to their nest entrance, as evidenced in our experiments but also in several ant species [1,2].
- Ants had a fixed probability to leave a food source $\tau = 0.01$ and spent an average time of 100 seconds on the food source, which is compatible with previous experimental observations (Lehue et al., in prep).
- When leaving the food source, ants laid a trail on their way back to the nest. In the model, a variable associated with each hexagonal cell represented the pheromone concentration in this cell, this concentration being incremented by each passage of a trail-laying ant by a value of 100.
- The local pheromone concentration influenced the path choice made by naive recruits. In the model, the agents were more likely to orient themselves towards the hexagonal cell with the highest concentration of pheromone (see Fig. SM1 below for a graphical representation of the movement of the simulated ants).

- The pheromone concentration of each hexagonal cell evaporated at a constant rate ν = 0.005. The choice of this value was guided by a previous paper by Collignon et al. 2010 on *Tetramorium caespitum* whose trail pheromone shares the same compound (3-ethyl-2,5-dimethylpyrazine) as the trail pheromone of *Myrmica rubra.*
- We simulated a total population of 90 foragers. This total population was equally distributed in subgroups among the different entrances out of which they could access the external environment and/or be recruited by successful foragers. Indeed, in our field experiments, the exit of recruits seemed to be independently activated at each nest entrance: a recruiter entering an entrance did not induce the mobilisation of workers at the neighbouring exits, even those belonging to the same cluster. Therefore, we simulated an independent recruitment process at each nest entrance (see Below).
- The exit rate of naive recruits increased non-linearly with the number of recruiters in the nest entrance. Previous work has evidenced the key role of tactile and chemical stimulation performed by the recruiters within the nest entrance [3,4] as well as the non-linear effects of the number of recruiting ants on the exit rate of recruits. The exit rate of naive ants at an entrance *i* was modelled by the function commonly used for recruitment [5,6]:

$$p_{exit\_i} = k\frac{\left(a + {R_i}^2\right)}{\left(b + {R_i}^2\right)}$$

Where R_i represents the number of recruiting ants inside the nest at the entrance *i*, *k* represents the maximal rate of exit, the ratio *ka*/*b* represents the probability for an ant to spontaneously exit the nest in the absence of any recruiter and the value of *a* and *b* (given a constant *a*/*b* ratio) allows the tuning of the effect of additional recruiters on the probability p_{exit_i} (large values of *a* and *b* reduce the impact of a recruiter meaning that for a given ratio, the largest the absolute value of a and b, the lower the impact of any additional recruiter on the exit rate of an individual). Here, we chose *k*=0.1, *a*=1, and *b*=300, so that in the absence of recruiting ants in the nest, each ant had a constant probability of 1/3,000 to leave the nest. Thus, for a population of 90 foragers, a spontaneous exit occurred approximately every 33.3 seconds. Here, we choose to use the number of

recruiters inside the nest entrance rather than the chemical concentration of the trail as the recruitment variable. This was chosen to account for the fact that the we mainly observed bursts of recruits exiting the nest after the return of a recruiter, rather than a continuous flow of exits. Therefore, the current equation is more representative of this process than the usual one based on the chemical trail whose recruiting effects are more spread out over time.

The model was run as follows:

At time 0, all the ants were naive and staying inside the nest. At each time step, naive ants had a probability p_e to spontaneously exit the nest and to search for food. The ants appeared on the foraging arena at the location of their dedicated nest entrance. At each time step, each ant that was on the foraging arena had a probability p_i to move to one of its neighbouring cells, which was computed as follows:

$$p_i = \frac{(d_i + q_i + m_i)^2}{\sum_{k=1}^{6}(d_k + q_k + m_k)^2}$$

With d_i, the bias towards cell i due to the current travelling direction of the ants (directional bias), q_i, the bias towards cell i due to the quantity of pheromone on cell i (trail-following bias), and m_i, the bias towards cell i due to the memory of the location of a food source or nest entrance (memory bias). This function is an extension of the one detailed in [7], with the inclusion of a directional bias and a memory bias. In the absence of any pheromone or memory, the searching pattern of each forager is thus a forward-biased random walk on a hexagonal grid (a simpler discretized version of the searching behaviour of foragers presented in [8]). Moreover, the introduction of a memory bias in the equation allowed us to reproduce the strong fidelity of the foragers to the first feeder that they discovered (as observed in our field experiments). Indeed, by setting the maximal value of the orientation bias (o_i), the steady state of the pheromone concentration of a well-marked trail (q_i), and the maximal value of the memory bias (m_i), so that $o_i < q_i \ll m_i$, we can simulate hierarchical decision rules in one equation. Indeed, naïve ants will be influenced by their current orientation but will reorient towards highly pheromone-concentrated cells (→ trail following behaviour), but informed ants will tend to return to the food source that they memorized, since the memory bias will

overrule the influence of their current orientation or the pheromones. At each time step and for each moving ant, a random number was drawn from a uniform distribution between 0 and 1 and a move was selected according to the probability to move towards each neighbouring cell. For an illustration of the movement of the ants on the grid, see the Fig. SM1 below while the main decision rules of the agents are summarized in the Table SM1 below.

Ants moved on the grid until they encountered by chance a cell bearing a food source (the food sources are not attractive). Once at a food source, an ant became informed about the location of this food source, thus determining its memory bias for the subsequent foraging outbound trips. After spending 100 time-steps at the feeder, ants returned to the nest carrying one unit of food and deposited a quantity of pheromone on each cell that they passed on the grid. Each returning ant tended to come back to its original nest entrance thanks to the memory bias associated with its own entrance. Once an ant arrived on the cell of its nest entrance, it unloaded its food item and started recruiting naive nestmates. At each time step, recruiting ants had a probability p=0.1 to exit the nest and go back to the food source that they had previously discovered. The fidelity of each ant for a specific entrance and food source was modelled by a memory bias that gave a higher weight to the cells forming the shortest path between the nest and the food source. By doing so, we assumed that ants were able to navigate straight between food resources and their home entrance, either by path integration and/or thanks to environmental cues. A table below (Table SM2) summarizes the main variables and parameters of the model.

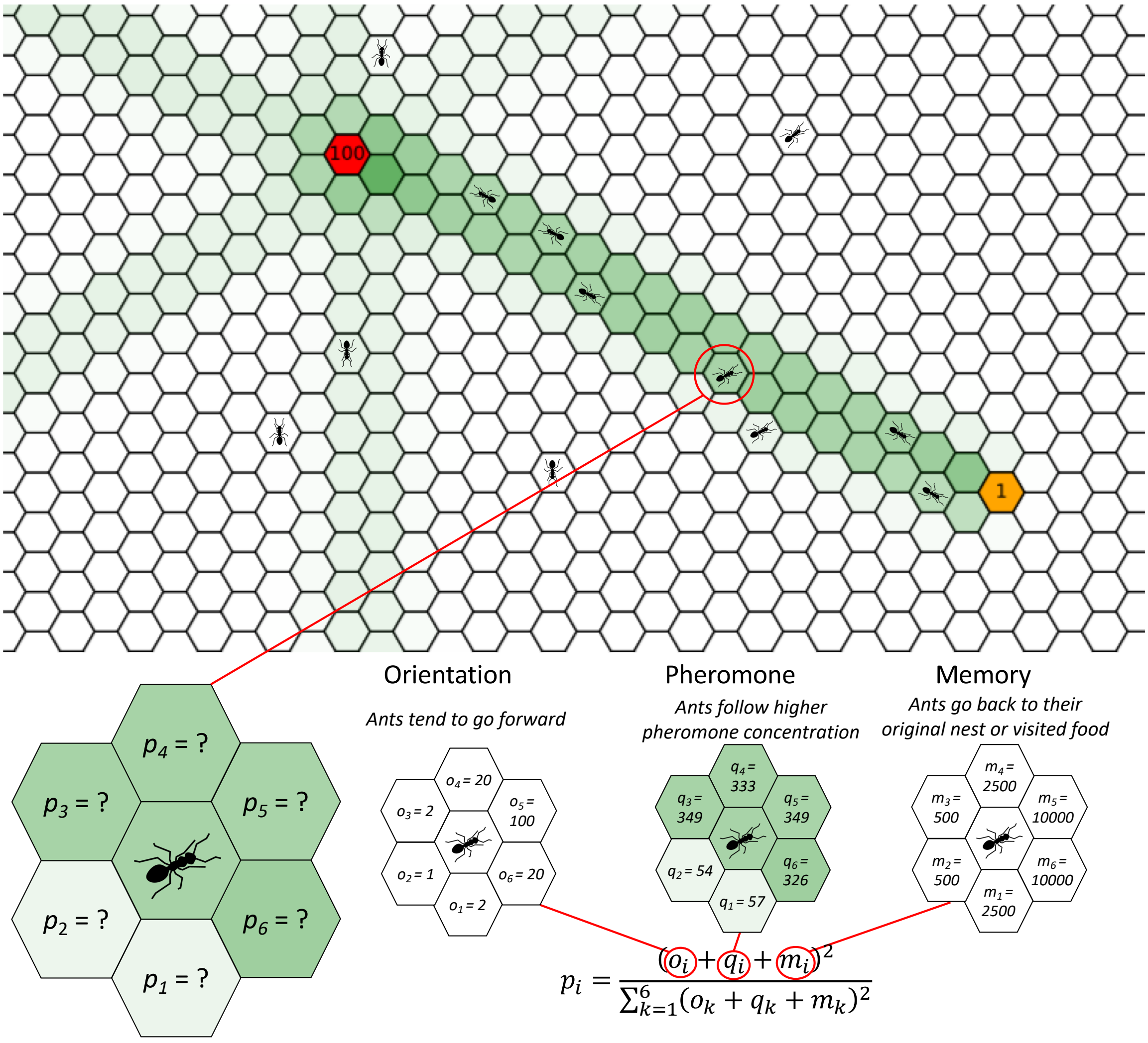

Fig. SM1. Simulation of the foraging activity of an ant colony. (Top) The nest entrance is represented in red (the number in the cell indicates the total number of foragers, 100 in this example), the food source is represented in orange (the number indicates the concentration of the sucrose solution, 1M in this example), the green shading represents the pheromone concentration on each cell (darker green indicates a stronger concentration). At each time step, each ant has a probability to move towards one of the six cells adjacent to its present position. (Bottom) Detail of the computation of the probability to move towards the ith cell. This probability is based on the current orientation of the ant (parameter o_i), the pheromone concentration of the adjacent cells (parameter q_i) and the memory of the ant (parameter m_i). The parameter's values were chosen so that the memory of the ants takes priority over the pheromone concentration which takes priority over the orientation of the ants. By doing so, (i) a naïve ant will mainly follow a straight path in the absence of pheromone; (ii) a naïve ant will follow a chemical trail if it is sufficiently reinforced; and (iii) an informed ant will mainly rely on its memory to come back to a previously visited food source or nest entrance.

Table SM1. Main decision rules of the agents according to their current state and position.

Current state of the agent	Action	Associated Probability
Naïve, in the nest	Stay in or leave the nest	p_{exit_i}
Recruiter, in the nest	Stay in or leave the nest	0.1
At a feeder	Stay or leave the feeder	0.01
Outside (but not at a feeder)	Move	p_i

Table SM2. Main variables and parameters of the model with their value (multiples instances indicate the different values that have been simulated).

Variables and parameters	Values
Number of nest entrances	1, 2, 3, 4, 6
Number of clusters of entrances	1, 2, 3, 4
Probability to leave the food source	0.01
Evaporation rate of the pheromone	0.005
Probability for a recruiter to leave the nest	0.1
k	0.1
a	1
b	300

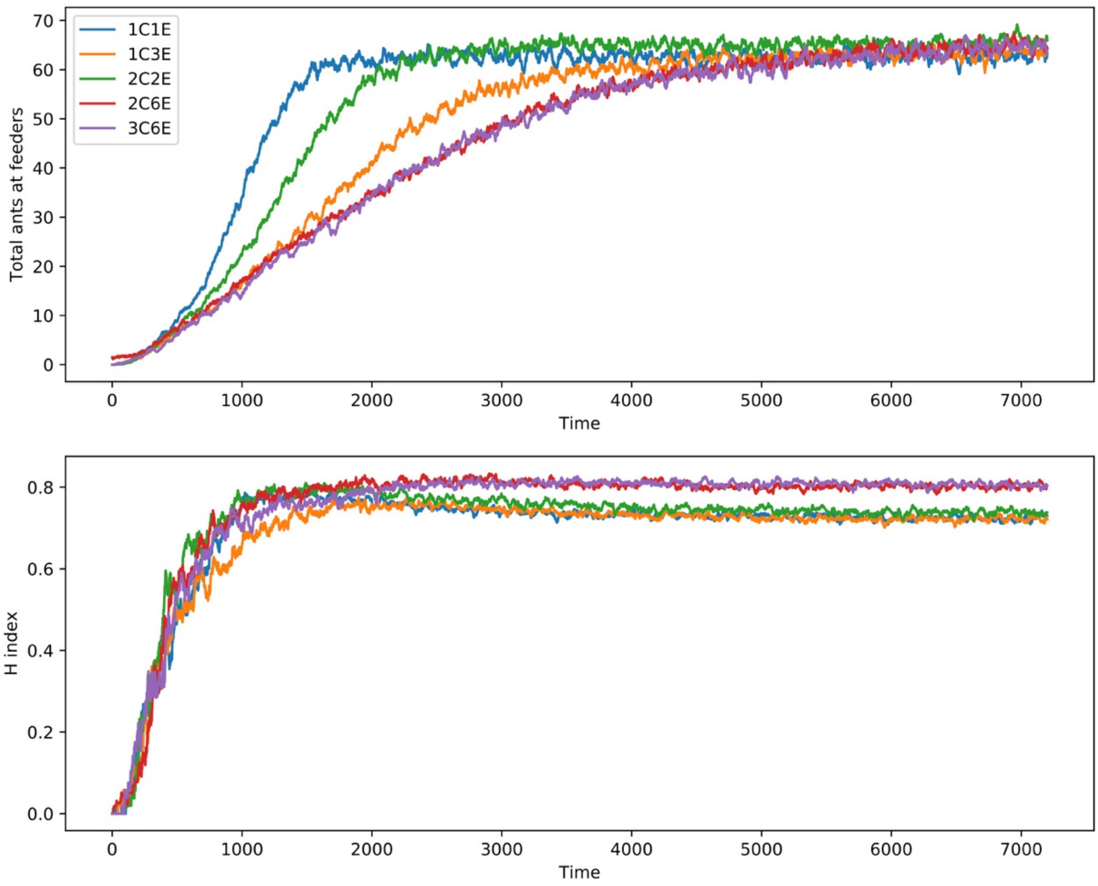

Figure SM2. *Theoretical dynamics of the exploitation of eight food sources on a hexagonal grid with each side of the hexagonal grid being 25-cell long, instead of 50.* (Upper panel) Total number of ants present on the food sources as a function of time. (Lower panel) Pielou's evenness indices accounting for the distribution of ants among the feeders as a function of time. In the colour legend, *XC* indicates the number of clusters and *XE* indicates the number of entrances. Overall, the same results are observed than for a larger grid.

References

1. Wehner R, Hoinville T, Cruse H, Cheng K. 2016 Steering intermediate courses : desert ants combine information from various navigational routines. *J. Comp. Physiol. A* **202**, 459–472. (doi:10.1007/s00359-016-1094-z)

2. Wystrach A, Mangan M, Webb B. 2015 Optimal cue integration in ants. *Proc. R. Soc. B Biol. Sci.* **282**.

3. de Biseau J-C, Pasteels JM. 2000 Response thresholds to recruitment signals and the

regulation of foraging intensity in the ant *Myrmica sabuleti* (Hymenoptera, Formicidae). *Behav. Processes* **48**, 137–148. (doi:10.1016/S0376-6357(99)00077-7)

4. Collignon B, Cervantes Valdivieso LE, Detrain C. 2014 Group recruitment in ants: who is willing to lead? *Behav. Processes* **108**, 98–104. (doi:10.1016/j.beproc.2014.09.033)

5. Beckers R, Deneubourg JL, Goss S. 1993 Modulation of trail laying in the ant *Lasius niger* (Hymenoptera: Formicidae) and its role in the collective selection of a food source. *J. Insect Behav.* **6**, 751–759. (doi:10.1007/bf01201674)

6. Collignon B, Deneubourg JL, Detrain C. 2012 Leader-based and self-organized communication: Modelling group-mass recruitment in ants. *J. Theor. Biol.* **313**, 79–86. (doi:10.1016/j.jtbi.2012.07.025)

7. Nicolis SC, Deneubourg J-L. 1999 Emerging Patterns and Food Recruitment in Ants : an Analytical Study. *J. Theor. Biol.* **198**, 575–592.

8. Cook Z, Franks DW, Robinson EJH. 2013 Exploration versus exploitation in polydomous ant colonies. *J. Theor. Biol.* **323**, 49–56. (doi:10.1016/j.jtbi.2013.01.022)
