# Supplementary material for: Nest Entrances, Spatial Fidelity, and Foraging Patterns in the Red Ant Myrmica rubra: A Field and Theoretical Study
Source: Insects. 2020 May 21;11(5):317. doi: 10.3390/insects11050317 (PMC7290572; doi:10.3390/insects11050317)
Supplement: Supplementary file 1 [file insects-11-00317-s001.zip › Supplementary/Figure_S3.pdf]

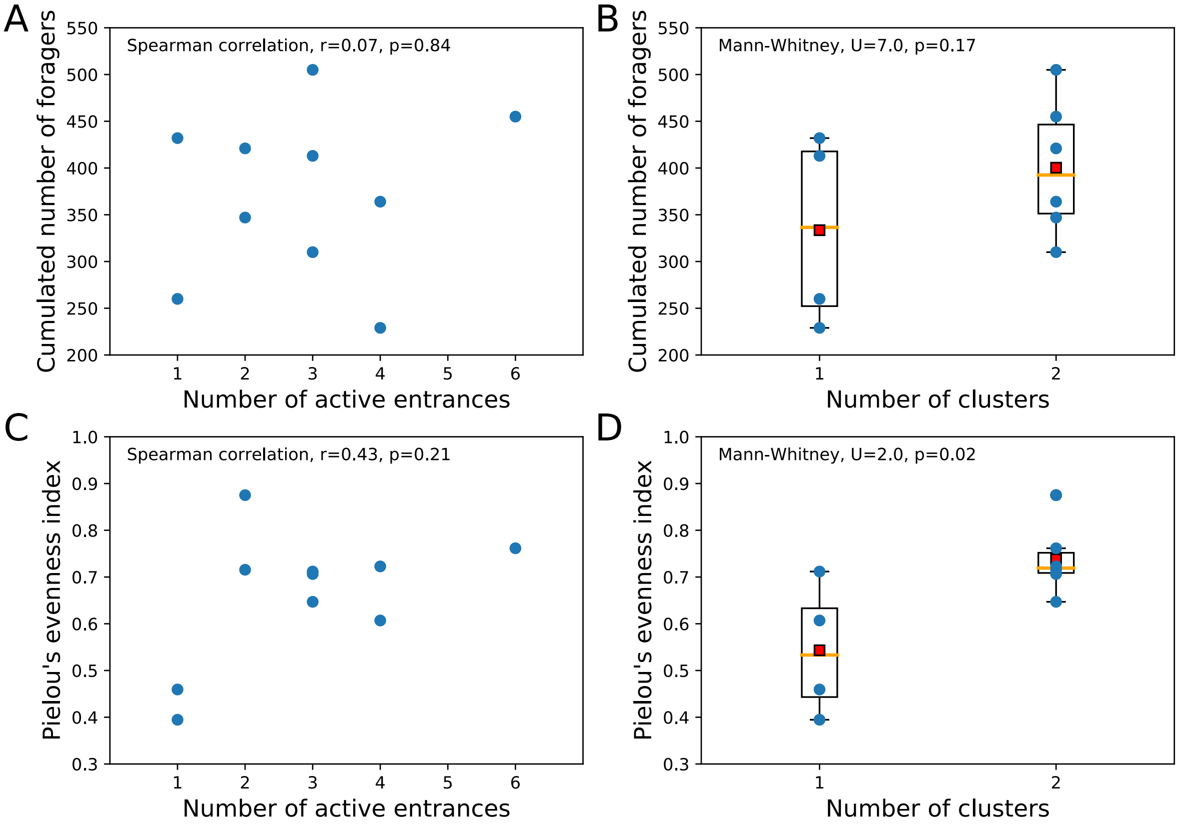

Fig. S3. *Influence of the number of entrances* (A, C) *and clusters* (B, D) *on the cumulated number of foragers observed at the feeders* (A,B) *and their distribution among the feeders quantified by the Pielou's evenness Index* (C,D). For each boxplot (C, D), the red square and the orange line represent the mean and the median of the data distribution, respectively.
