# Supplementary material for: Nest Entrances, Spatial Fidelity, and Foraging Patterns in the Red Ant Myrmica rubra: A Field and Theoretical Study
Source: Insects. 2020 May 21;11(5):317. doi: 10.3390/insects11050317 (PMC7290572; doi:10.3390/insects11050317)
Supplement: Supplementary file 1 [file insects-11-00317-s001.zip › Supplementary/Figure_S2.pdf]

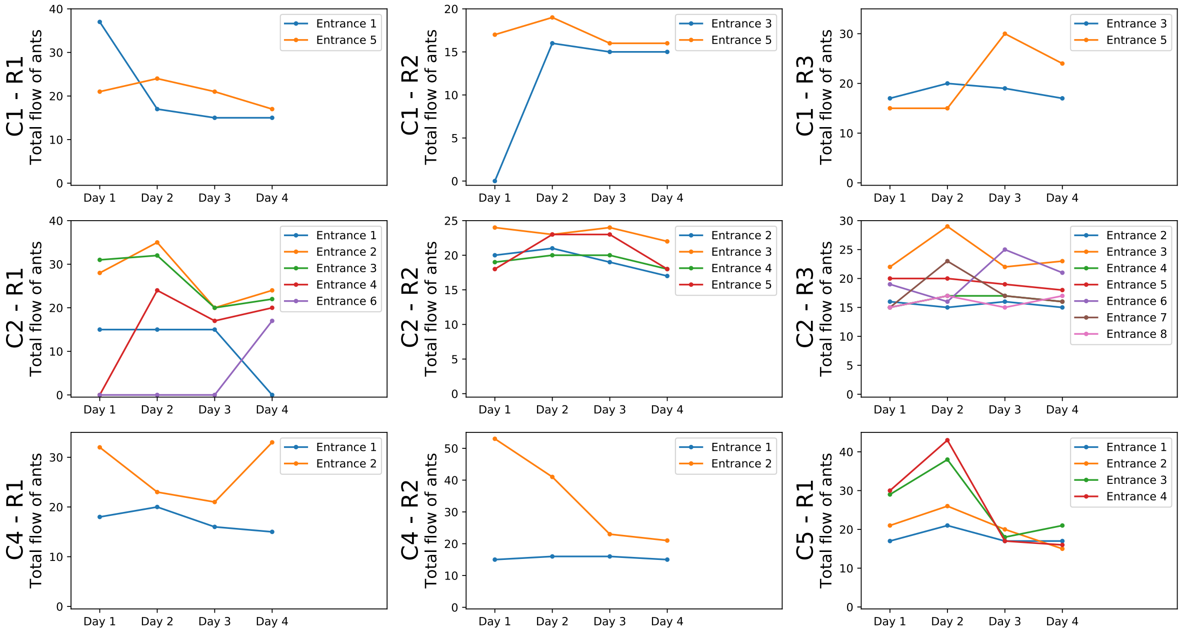

Fig. S2 *Monitoring of the flows of workers at each nest entrance*. For four consecutive days, we monitored for 10 minutes the total (in and out) flow of workers at each entrance. We monitored all entrances located at Day 1 of the observation session. Colony 3 is not included in the analysis since the colony moved out after two days of monitoring.
