# Supplementary material for: Nest Entrances, Spatial Fidelity, and Foraging Patterns in the Red Ant Myrmica rubra: A Field and Theoretical Study
Source: Insects. 2020 May 21;11(5):317. doi: 10.3390/insects11050317 (PMC7290572; doi:10.3390/insects11050317)
Supplement: Supplementary file 1 [file insects-11-00317-s001.zip › Supplementary/Figure_S1.pdf]

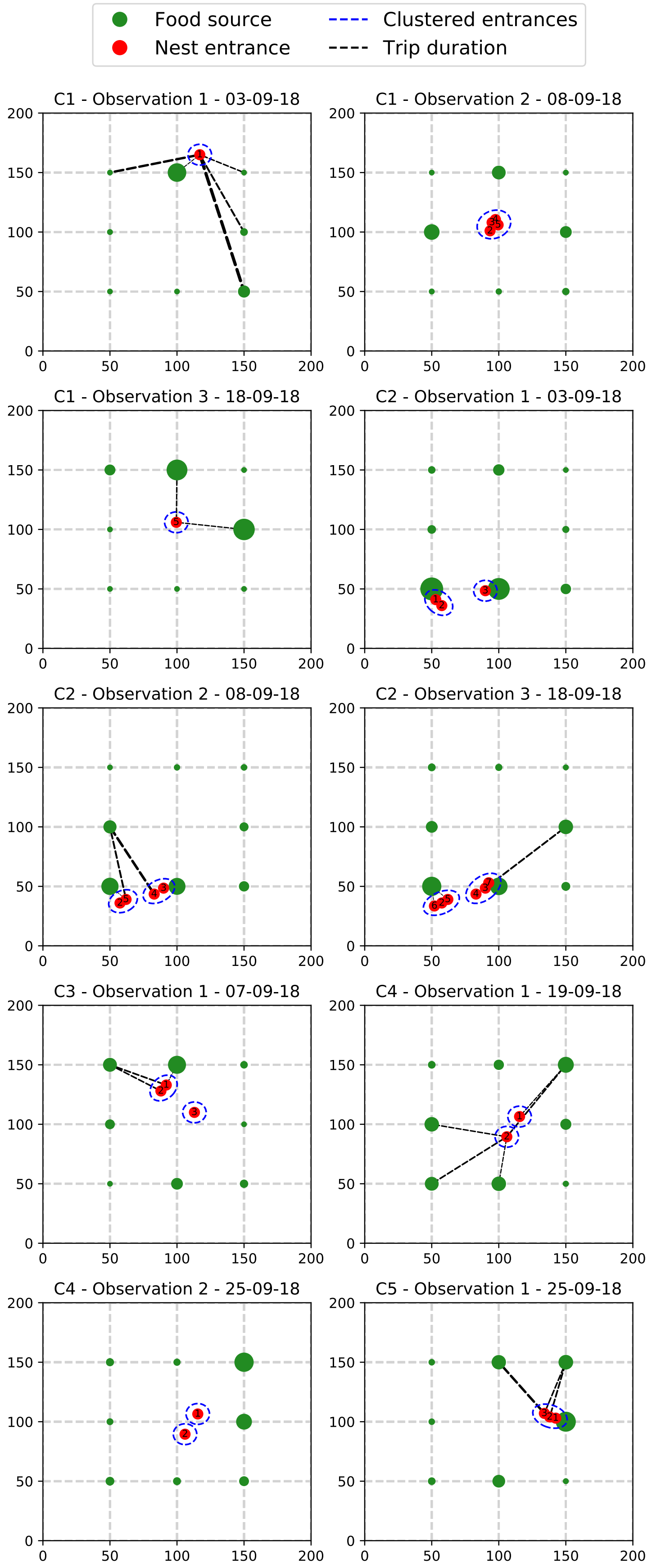

Fig. S1 *Spatial distribution of the entrances of Myrmica rubra colonies in 4m^2 quadrats.* Each red dot corresponds to an entrance. Green dots represent the eight feeders. The size of the green dots is proportional to the cumulated number of foragers that visited a feeder during the experiment. Dashed black lines represent the trip duration between feeders and entrances. The line thickness is proportional to the trip duration averaged over 10 foraging journeys. Dashed blue circles represent clusters of entrances.
