# Supplementary material for: Nest Entrances, Spatial Fidelity, and Foraging Patterns in the Red Ant Myrmica rubra: A Field and Theoretical Study
Source: Insects. 2020 May 21;11(5):317. doi: 10.3390/insects11050317 (PMC7290572; doi:10.3390/insects11050317)
Supplement: Supplementary file 1 [file insects-11-00317-s001.zip › Supplementary/Figure_S5.pdf]

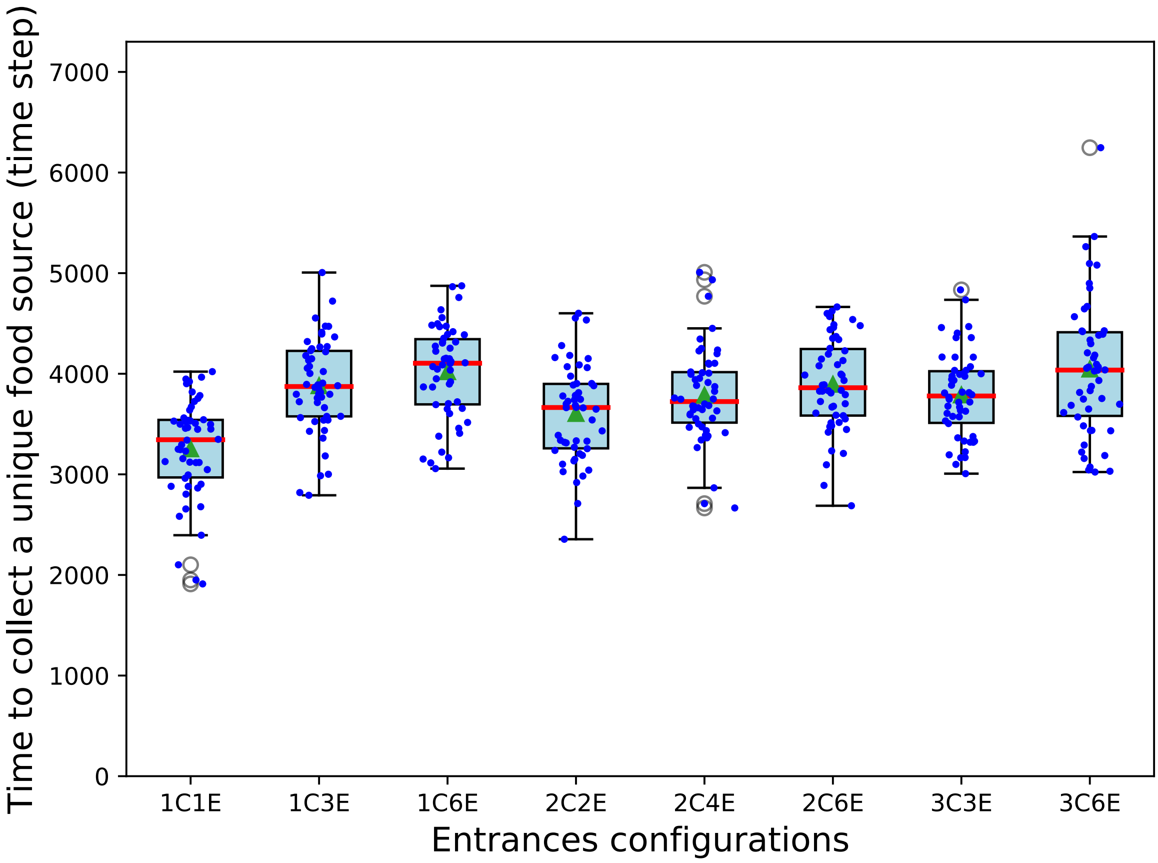

Fig. S5 *Influence of the type of entrance clustering on the timing required to collect a unique food source of 600 units*. In the legend, C indicates the number of clusters and E the total number of entrances (equally distributed among the clusters). Green triangles indicate the median, red lines indicate the mean and circles indicate outliers.
