# Supplementary material for: Nest Entrances, Spatial Fidelity, and Foraging Patterns in the Red Ant Myrmica rubra: A Field and Theoretical Study
Source: Insects. 2020 May 21;11(5):317. doi: 10.3390/insects11050317 (PMC7290572; doi:10.3390/insects11050317)
Supplement: Supplementary file 1 [file insects-11-00317-s001.zip › Supplementary/Figure_S4.pdf]

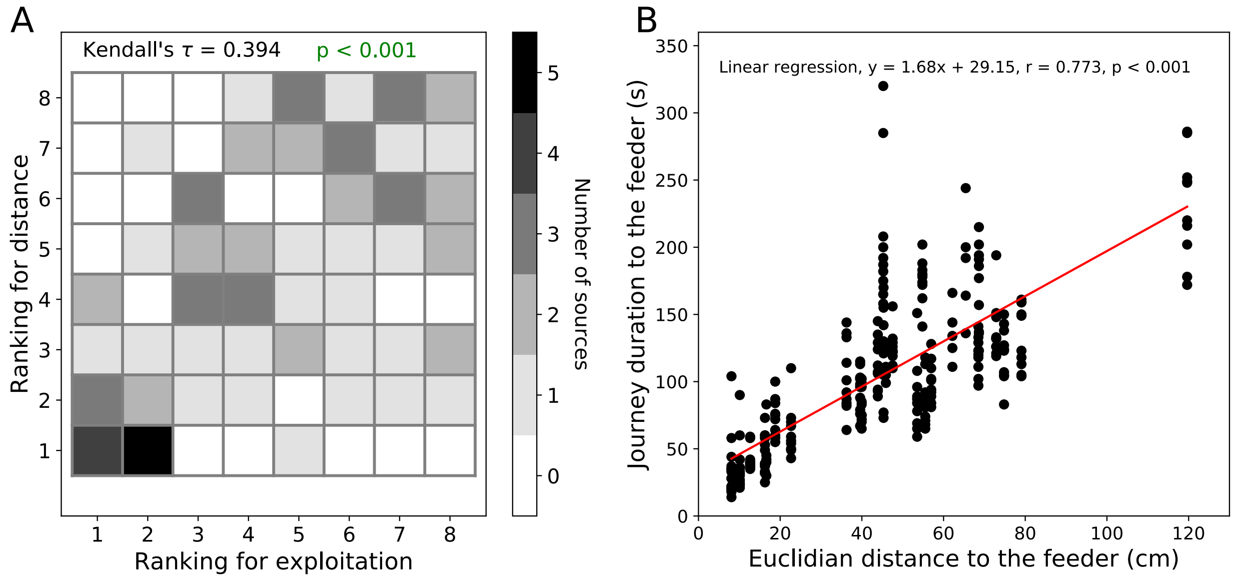

Fig. S4 *Influence of entrance proximity on the exploitation of a food source*. (A) For each observation, each food source was ranked according to two criteria: its proximity to the closest entrance, and its level of exploitation (i.e. the cumulated number of ants observed at this feeder every 15 minutes and cumulated over the 2-hour observation period). Correlation between the two rankings was tested (Kendall tau test, p<0.001). (B) Trip duration between the feeder and the nest entrance according to the Euclidian distance between the feeder and the nest entrance.
